# Gestational weight gain information: seeking and sources among pregnant women

**DOI:** 10.1186/s12884-015-0600-6

**Published:** 2015-08-07

**Authors:** Jane C. Willcox, Karen J. Campbell, Elizabeth A. McCarthy, Martha Lappas, Kylie Ball, David Crawford, Alexis Shub, Shelley A. Wilkinson

**Affiliations:** Centre for Physical Activity and Nutrition Research, Deakin University, 221 Burwood Hwy, Burwood, Victoria 3125 Australia; Department of Obstetrics and Gynaecology, University of Melbourne/Mercy Hospital for Women, Melbourne, Australia; Mater Research Institute, University of Queensland, South Brisbane, Queensland Australia; Department of Nutrition & Dietetics, Mater Mothers Hospital, South Brisbane, Queensland Australia

**Keywords:** Information seeking behaviour, Pregnancy, Gestational weight gain, Weight management

## Abstract

**Background:**

Promoting healthy gestational weight gain (GWG) is important for preventing obstetric and perinatal morbidity, along with obesity in both mother and child. Provision of GWG guidelines by health professionals predicts women meeting GWG guidelines. Research concerning women’s GWG information sources is limited. This study assessed pregnant women’s sources of GWG information and how, where and which women seek GWG information.

**Methods:**

Consecutive women (*n* = 1032) received a mailed questionnaire after their first antenatal visit to a public maternity hospital in Melbourne, Australia. Recalled provision of GWG guidelines by doctors and midwives, recalled provided GWG goals, and the obtaining of GWG information and information sources were assessed.

**Results:**

Participants (*n* = 368; 35.7 % response) averaged 32.5 years of age and 20.8 weeks gestation, with 33.7 % speaking a language other than English. One in ten women recalled receiving GWG guidelines from doctors or midwives, of which half were consistent with Institute of Medicine guidelines. More than half the women (55.4 %) had actively sought GWG information. Nulliparous (OR 7.07, 95 % CI = 3.91–12.81) and obese (OR 1.96, 95 % CI = 1.05–3.65) women were more likely to seek information. Underweight (OR 0.29, 95 % CI = 0.09–0.97) women and those working part time (OR 0.52, 95 % CI = 0.28–0.97) were less likely to seek information. Most frequently reported GWG sources included the internet (82.7 %), books (55.4 %) and friends (51.5 %). The single most important sources were identified as the internet (32.8 %), general practitioners (16.9 %) and books (14.9 %).

**Conclusion:**

More than half of women were seeking GWG guidance and were more likely to consult non-clinician sources. The small numbers given GWG targets, and the dominance of non-clinical information sources, reinforces that an important opportunity to provide evidence based advice and guidance in the antenatal care setting is currently being missed.

**Electronic supplementary material:**

The online version of this article (doi:10.1186/s12884-015-0600-6) contains supplementary material, which is available to authorized users.

## Background

Achieving healthy gestational weight gain (GWG) is acknowledged as important in promoting positive maternal and fetal health outcomes in both the short and long term [1]. Weight gained in excess of GWG guidelines, termed ‘excess GWG’, is associated with increased prevalence of gestational diabetes, hypertensive disorders, delivery complications, large for gestational age, and the long term risk of obesity for both mother and child [2–5]. The 2009 Institute of Medicine (IOM) guidelines [1] are most commonly adopted in developed countries in the absence of country specific guidelines [6] and are central to the recent refocus on GWG.

Consistent with the increased prevalence of overweight and obesity in women in their childbearing years in developed countries [7], there is also an increasing prevalence of excess GWG [1, 8]. Between 35–60 % of women, across all body mass index (BMI) categories, exceed the recommended guidelines [2, 7, 9]. Women who are overweight or obese at conception are most at risk of exceeding GWG guidelines [10]. However, women’s understanding of appropriate GWG and the consequences for maternal and child health of excess GWG is generally poor [11]. Women view pregnancy as a time when weight is gained and may be retained or lost post-partum [11, 12].

Studies indicate that the provision of GWG guidelines by health professionals and knowledge regarding appropriate GWG is predictive of meeting GWG guidelines [13, 14]. A recent study of 249 US women reported that having health providers offer IOM weight gain recommendations increased the likelihood of women setting a concordant GWG goal (vs. no goal) (OR = 5.3, 95 % CI: 1.5, 18.6), which in turn was predictive of actual weight gains that fell within IOM guidelines [14]. These findings highlight the importance of women and their health service providers identifying and understanding GWG targets.

Very little is known about GWG information seeking behaviours of women, despite pregnancy being a time of significant information seeking and knowledge acquisition for women [15]. Qualitative studies suggest that women are dissatisfied with the level of health professionals’ provision of GWG information and that they may be seeking their information elsewhere [16, 17]. Whilst it is understood that women will attain pregnancy related information from a range of sources, including books, magazines, the internet, family, friends and health professionals [18], there is currently little evidence concerning the GWG related information sources women are accessing.

The acquisition of GWG information is an important early step in achieving a healthy weight in pregnancy [19]. When planning healthy GWG interventions, a baseline understanding of potential influences on pregnant women’s GWG information seeking and sources is required to allow effective targeting for knowledge and behaviour change. To our knowledge, no other studies have examined women’s GWG information sources and seeking nor their demographic predictors in detail. Therefore, this study aimed to investigate pregnant women’s GWG information sources and information seeking and how they varied by key demographic factors.

## Methods

A cross sectional study, Pregnancy, Health, Information and You (PHIY), utilising a mailed self-administered survey was carried out at a major Australian maternity tertiary training hospital with eligibility screening between October 2012 and January 2013. The hospital did not have protocols for regular weighing or GWG counselling. The PHIY study was designed to explore GWG attitudes, knowledge and pregnancy information seeking behaviours in pregnant women at the time of their first hospital visit. The data utilised in this study were derived from the PHIY. Ethics approval was obtained from Deakin University (2012–183) and Mercy Hospital for Women (R12/29) Human Research Ethics Committees.

### Study population

Consecutive eligible pregnant women were recruited from public antenatal clinic attendance registers following the woman’s first hospital antenatal visit. Inclusion criteria included sufficient English to complete the survey, aged more than 18 years and continuing pregnancy care at the hospital.

### Recruitment

Study packages were mailed to eligible women. The study package contained: introductory letter; pen and paper survey; plain language statement; consent form; and return stamped envelope. Participants were followed up using a protocol informed by the total design method as described by Dillman [20]. Non-responders were followed up two weeks after the initial mail-out with a reminder letter, and three weeks after the initial mail-out with a replacement questionnaire.

### Survey design

The survey question development was informed by the literature and discussion with a wide range of health professionals working with pregnant women. Constructs reported in this study reflected understandings of the correlates of healthy GWG [9, 14, 21], the predictors of health behaviours [19] and the ‘uses and gratification’ theory, a theory of media usage [22]. The uses and gratification theory assumes that users deliberately choose media that will satisfy a given need. The survey instrument was pilot tested for comprehensibility and clarity with a convenience sample of ten pregnant or recently pregnant women. Minor amendments were made based on this process.

The researchers did not have access to participants’ email addresses and thus a hard copy mailed survey was chosen. Support for this approach is provided by literature that suggests that mailed surveys may achieve a higher response rate and produce a higher internal consistency than web or internet surveys [23, 24]. To encourage completion of potentially sensitive information, anonymity for the surveys was guaranteed.

### Measures

#### Background characteristics

Pregnancy characteristics assessed comprised of parity and due date. Socio-demographic variables assessed included: maternal date of birth; relationship status; country of birth; and primary language. Maternal education was categorised in three levels: completion of secondary education or less; trade/certificate or diploma; and tertiary education. Main daily activity was defined in five categories: working fulltime; working part time; raising children; studying fulltime; and unemployed. Per annum household income was divided into five categories: <$51,999; $52-77,999; $78-99,999; >$99,999; and an option not to answer. Self-reported access to internet and ownership of mobile phones were dichotomous indicators of technology usage. Anthropometric questions included self-reported pre-pregnancy height and weight which were used to calculate pre-pregnancy BMI (ppBMI). BMI categories were defined based on the World Health Organization guidelines [25].

Basic demographic data, including age (as a continuous variable) and primary language, were collected from the hospital data base on all eligible women allowing comparisons between responders and non-responders.

### Outcome variables

Constructs related to GWG information included:

1. GWG information seeking;
2. Information sources accessed for GWG information; and
3. Recalled GWG guideline provision by a doctor or midwife.

The latter assessed participant recall of the counselling rather than health professional report because ultimately the participant’s own recall will influence weight gain and the advice provided was unavailable. Additional file 1 outlines the questions, sources and reliability testing of the questions.

Reliability testing was conducted on questions that had not been tested previously. Survey question test-retest reliability was established via repeated administration of the survey two weeks apart in a separate sample of 38 pregnant women.

### Sample size

The outcome measure utilised for sample size calculations was the provision of GWG guidelines by a doctor or midwife, a dichotomous variable. A study sample of *n* = 360 from a potential population of 5500 allowed estimation with 95 % confidence intervals of a population proportion being provided with GWG guidelines with precision of approximately 5 % and estimation of a population mean with precision of approximately 10 % of the population standard deviation.

### Data analyses

Analysis was conducted using Stata 12 (Stata Corp, College Station, Texas, USA). Descriptive statistics (mean, standard deviation, percent and range) were used to describe characteristics of the sample. The normality of continuous variables was assessed by visual inspection of histograms. Statistical tests (group mean comparison t test and χ^2^ test) were used to assess differences in characteristics between non responders and responders, identified by hospital identification number to maintain anonymity. Logistic regression was used to investigate the predictors of GWG information seeking and GWG sources nominated as the most important, controlling for technology usage. Associations between each individual characteristic (socio-demographic status, pregnancy, ppBMI and technology usage) and GWG information seeking were identified in bivariable logistic regression analyses, and only those characteristics significantly (p ≤ 0.05) associated with outcome were adjusted for in multivariable analyses. This procedure was repeated to identify predictors for the nomination of the most important GWG information sources grouping sources into three categories of media (internet, brochures, books, blogs, magazines), health professionals and family and friends.

## Results

**Fig. 1:**
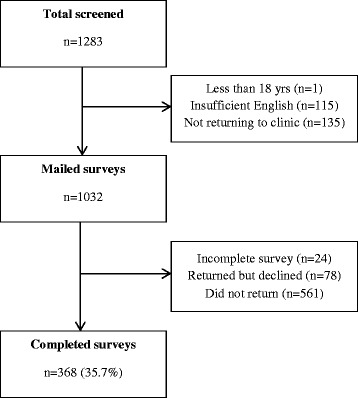
Flow diagram of study recruitment

A total of 1032 consecutive eligible women received a mailed questionnaire after their first antenatal visit. Thirty six percent of participants completed surveys that were eligible for inclusion (*n* = 368) (Fig. 1). Responders were significantly older (32.5 vs 31.1 years, p < 0.001) than the non-responders but with no significant difference reported for a primary language other than English.

### Participant characteristics

The characteristics of the 368 participants are presented in Table 1. The mean age of participants was 32.5 years with a mean gestation of 20.8 weeks. Nearly one half of women were nulliparous. More than one third of women were born overseas and spoke a language other than English at home. More than half of the women had some tertiary education. Approximately one third of women reported working full time with another third working part time. More than one half reported a household income over $78,000. The majority of participants had access to the internet and a mobile phone. Twenty percent of the women had a BMI in the overweight and 15.2 % in the obese ranges.

**Table 1:** Participant characteristics

Characteristics (*n* = 368)	Mean (SD)
Gestation (weeks)	20.8 (5.5)
Gestation at first clinic visit (weeks)	16.1 (4.3)
	n (%)
Maternal age (mean 32.5 years, SD 4.5)	
≤25 years	20 (5.4)
26–30 years	142 (38.6)
31–35 years	119 (32.3)
≥36 years	87 (23.6)
Country of birth	
Australia	230 (62.5)
Overseas	138 (37.5)
Aboriginal/Torres Strait Islander	
Yes	1 (0.3)
No	367 (99.7)
Language	
English	244 (66.3)
LOTE	124 (33.7)
Relationship status	
Married/defacto	360 (97.8)
Separated/widowed/never married	8 (2.2)
Education	
Secondary or less	40 (10.9)
Trade/Certificate or Diploma	101 (27.5)
Tertiary	227 (61.7)
Main daily activity	
Working full time	137 (37.2)
Working part time	118 (32.1)
Raising children	92 (25.0)
Unemployed	13 (3.5)
Studying full time	8 (2.2)
Household income	
<$51,999	72 (19.6)
$52–77,999	64 (17.4)
$78–99,999	72 (19.6)
>$99,999	109 (29.6)
Did not answer	51 (13.9)
Single/multiple pregnancy	
Single	360 (97.8)
Multiple	8 (2.2)
Parity	
0	173 (47.0)
≥1	195 (53.0)
BMI kg/m^2^ (mean 24.6, SD 5.6)	
Underweight (<18.5 kg/m^2^)	18 (4.9)
Healthy weight (18.5–24.9 kg/m^2^)	219 (59.5)
Overweight (25–29.9 kg/m^2^)	75 (20.4)
Obese (30–34.9 kg/m^2^)	56 (15.2)
Use internet	
Yes	363 (99.2)
No	3 (0.8)
Mobile phone ownership	
Yes	362 (98.6)
No	5 (1.4)

*BMI* body mass index, *LOTE*, language other than English, *SD* standard deviation

### GWG information seeking

More than half of the women (*n* = 202, 54.9 %) had actively sought GWG information. Table 2 presents the results of the bivariable and multivariable logistic regression analysis of GWG information seeking. In the multivariable analysis, four variables remained significant predictors of GWG information seeking. Women having their first child were seven times more likely to seek GWG information than those with subsequent pregnancies. Those with a BMI in the obese category were more than twice as likely to seek GWG information compared with women in the healthy weight range. Women nominating “working part time” as their key daily activity were half as likely to seek GWG information as those who nominated raising children fulltime. Those with a BMI in the underweight category were two thirds less likely to seek information compared with women in the healthy weight range.

**Table 2:** Odds ratios and 95 % confidence intervals from bivariable and multivariable logistic regression analyses predicting GWG information seeking

*n* = 368	Sought GWG information		Bivariable associations^a^			Multivariable associations^a,b^		
	No (n/%)	Yes (n/%)	OR	95 % CI	p	OR	95 % CI	p
Total	166	202						
Maternal age								
≤30 years	65 (39.2)	97 (48.0)	1.00	Ref		1.00	Ref	
31–35 years	62 (37.3)	57 (28.2)	0.62	0.38, 0.99	0.05	1.15	0.64, 2.07	0.63
≥36 years	39 (23.5)	48 (23.8)	0.83	0.45, 1.4	0.47	1.45	0.76, 2.77	0.26
Country of birth								
Australia	110 (66.3)	120 (59.4)	1.00	Ref				
Overseas	56 (33.7)	82 (40.6)	1.35	0.88, 2.05	0.18			
Language								
English	114 (68.7)	130 (64.4)	1.00	Ref				
LOTE	52 (31.3)	72 (35.6)	1.21	0.78, 1.88	0.87			
Education								
Secondary or less	22 (13.3)	18 (8.9)	1.00	Ref				
Trade/Certificate/Diploma	50 (30.1)	51 (25.2)	1.25	0.60, 2.60	0.56			
Tertiary	94 (56.6)	133 (65.9)	1.73	0.88, 3.40	0.11			
Main daily activity								
Working full time	39 (23.5)	98 (48.5)	2.89	1.65, 5.00	<0.01	0.79	0.39, 1.60	0.52
Working part time	68 (41.0)	50 (24.8)	0.84 (p < 0.001)	0.48, 1.45	0.53	0.52	0.28, 0.97	0.04
Raising children	49 (29.5)	43 (21.3)	1.00	Ref		1.00	Ref	
Unemployed/studying	10 (6.0)	11 (5.4)	1.25	0.49, 3.24	0.64	0.40	0.13, 1.24	0.11
Household income								
<$51,999	37 (22.3)	35 (17.3)	1.00	Ref		1.00	Ref	
$52–77,999	34 (20.5)	30 (14.9)	0.98	0.52, 1.83	0.84	0.86	0.40, 1.85	0.71
$78–99,999	37(22.3)	35 (17.3)	1.00	0.52, 1.91	1.00	1.09	0.51, 2.31	0.83
>$99,999	37(22.3)	72 (35.6)	2.01	1.11, 3.78	0.02	1.96	0.96, 4.01	0.07
Did not answer	21(12.6)	30 (14.9)	1.51	0.73, 3.12	0.27	1.57	0.69, 3.55	0.28
Parity								
0	41 (24.7)	132 (65.3)	5.78	3.64, 9.08	<0.01	7.07	3.91, 12.81	<0.01
≥1	125 (75.3)	70 (34.7)	1.00	Ref		1.00	Ref	
BMI kg/m^2^								
Underweight (<18.5 kg/m^2^)	13 (7.8)	5 (2.5)	0.32	0.11, 0.92	0.04	0.29	0.09, 0.97	0.04
Healthy weight (18.5–24.9 kg/m^2^)	99 (59.6)	120 (59.4)	1.00	Ref		1.00	Ref	
Overweight (25–29.9 kg/m^2^)	37 (22.3)	38 (18.8)	0.85	0.50, 1.43	0.54	1.02	0.56, 1.87	0.94
Obese (30–34.9 kg/m^2^)	17 (10.2)	39 (19.3)	1.89	1.01, 3.55	0.05	2.61	1.23, 5.33	0.01

*Ref*., reference category; *LOTE*, language other than English

^a^ Analysis adjusted for access to technology

^b^ Characteristics significantly (p ≤ 0.05) associated with outcome in bivariable analysis were included in multivariable analyses

### Recalled provision of GWG guidelines by health professionals

Only 9.5 % (*n* = 35) of women recalled receiving GWG guidelines from doctors or midwives. Of the recalled weight guidelines given by health professionals, 54.3 % (*n* = 19) were consistent with IOM GWG guidelines. The small numbers in this group precluded further analysis of predictors.

### Sources of GWG information

Women reported accessing multiple sources for GWG information (Table 3). The most commonly accessed sources included the internet (82.7 %), books (55.4 %), friends (51.5 %), general practitioner (GP; 44.6 %) and family (43.1 %). When asked to nominate their most important source of GWG information one third of women cited the internet as their most important source (32.8 %) followed by GPs (16.9 %), books (14.9 %) and obstetricians (12.3 %).

**Table 3:** Sources of GWG information

Source of GWG information (*n* = 202)	Sources accessed^a^ (n/%)	Single most important source^b^ (n/%)
Internet	167 (82.7)	64 (32.8)
Books	112 (55.4)	29 (14.9)
Friends	104 (51.5)	9 (4.6)
GP	90 (44.6)	33 (16.9)
Family	87 (43.1)	4 (2.1)
Magazines	74 (36.6)	3 (1.5)
Midwife	58 (28.7)	15 (7.7)
Obstetrician	51 (25.2)	24 (12.3)
Chat/blog	32 (15.8)	2 (1.0)
Brochures	30 (14.9)	3 (1.5)
Dietitian	20 (9.9)	8 (4.1)
Social media	10 (5.0)	
TV/Radio	7 (3.5)	
Other	6 (3.0)	1 (0.5)
Naturopath	2 (1.0)	
Pharmacist	2 (1.0)	

*GP*, General Practitioner or primary care physician

^a^ Multiple sources allowed

^b^ *n* = 195 responses

Further analysis was conducted on the two groups of women choosing health professionals or media (internet, brochures, books, blog, magazines) as their most important source of information regarding GWG. The small numbers choosing family and friends precluded analyses. Table 4 presents the results of the bivariable and multivariable logistic regression analyses predicting the choice of health professionals or media as the most important source utilising health professionals as the reference category. After controlling for the two significant bivariable variables in the multivariable analysis, country of birth remained the only significant predictor. Women born in a country other than Australia were three times more likely than those born in Australia to view media sources as the most important GWG information source.

**Table 4:** Odds ratios and 95 % confidence intervals from bivariable and multivariable logistic regression analysis predicting use of health professionals or media as the most important GWG source^a^

*n* = 181	Sought GWG information		Bivariate associations^b^			Multivariate associations^b,c^		
	Health prof (n/%)	Media (n/%)	OR	95 % CI	p	OR	95 % CI	p
Total	80	101						
Maternal age								
≤30 years	35 (43.7)	55 (54.5)	1.00	Ref				
31–35 years	23 (28.8)	27 (26.7)	0.75	0.37, 1.50	0.41			
≥36 years	22 (27.5)	19 (18.8)	0.83	0.26, 1.16	0.12			
Country of birth								
Australia	59 (73.8)	48 (47.5)	1.00	Ref		1.00	Ref	
Overseas	21 (26.2)	53(52.5)	3.10	1.65, 5.84	<0.01	3.20	1.76, 6.85	<0.01
Language								
English	58 (72.5)	60 (59.4)	1.00	Ref				
LOTE	22 (27.5)	41 (40.6)	1.80	0.96, 3.39	0.07			
Education								
Secondary or less	9 (11.2)	6 (5.9)	1.00	Ref				
Trade/Certificate Diploma	17 (21.3)	28 (27.7)	2.47	0.75, 8.17	0.14			
Tertiary	54 (67.5)	67 (66.3)	1.73	0.88, 3.40	0.28			
Main daily activity								
Working full time	35 (43.7)	52 (51.5)	0.69	0.31, 1.51	0.34	1.02	0.44, 2.37	0.96
Working part time	27 (33.8)	17 (16.8)	0.29	0.12, 0.72	0.01	0.40	0.16, 1.03	0.06
Raising children	13 (16.3)	28 (27.7)	1.00	Ref		1.00	Ref	
Unemployed/studying	5 (6.2)	4 (4.0)	0.37	0.09, 1.62	0.19	0.34	0.07, 1.57	0.17
Household income								
<$51,999	12 (15.0)	17 (16.8)	1.00	Ref				
$52–77,999	11 (13.8)	17 (16.8)	1.10	0.38, 3.15	0.87			
$78–99,999	13 (16.3)	18 (17.8)	0.98	0.35, 2.73	0.97			
>$99,999	29 (36.2)	37 (12.9)	0.90	0.38, 2.12	0.82			
Did not answer	15 (18.7)	12 (11.8)	0.56	0.20, 1.63	0.29			
Parity								
0	49 (61.3)	70 (69.3)	1.43	0.77, 2.65	0.26			
≥1	31 (38.7)	31 (30.7)	1.00	Ref				
BMI kg/m^2^								
Underweight (<18.5 kg/m^2^)	1 (1.3)	3 (3.0)	2.11	0.21, 21.02	0.52			
Healthy weight (18.5-24.9 kg/m^2^)	43 (53.8)	61 (60.4)	1.00	Ref				
Overweight (25–29.9 kg/m^2^)	13 (16.2)	21 (20.8)	1.14	0.51, 2.52	0.75			
Obese (30–34.9 kg/m^2^)	23 (28.7)	16 (15.8)	0.49	0.23, 1.04	0.06			

*Ref*. reference category, *LOTE* language other than English

^a^ Reference category – health professional

^b^ Analysis adjusted for access to technology

^c^ Characteristics significantly (p ≤ 0.05) associated with outcome in bivariable analysis were included in multivariable analyses

## Discussion

This study showed that in this sample of women attending a public maternity hospital, more women were likely to seek GWG information than be offered GWG guidelines by health professionals. The small numbers (9.5 %) provided with GWG guidelines and with evidence based guidelines (5.2 %) was lower than figures reported in similar studies [16]. For example, a recent study of practitioner provision of healthy GWG advice to 310 Canadian women reported that 28.5 % of women were counselled correctly by their health care providers about GWG [16]. While it must be acknowledged that reported provision of GWG guidelines from health professionals was unavailable, the small numbers of women recalling guidelines in the present study is concerning given evidence suggesting that provision of guidelines increases the likelihood of women setting a concordant GWG goal and gaining weight consistent with the guidelines [14].

The low rate of guideline provision reported in this study may be at least partly attributable to the cross sectional study design. Surveying women at various time points across pregnancy may have produced different results given that information requirements may potentially change over the course of pregnancy. Moreover, health professionals often raise issues when a problem is noted [26]. Conversely, the average gestation of women completing the study was 20.8 weeks and women would likely have attended a minimum of two health professional visits, one with their general practitioner in the community and one at the antenatal clinic, and most likely more. That the majority of women, more than half way through their gestation, have not recalled receiving GWG guidelines raises concern and signals opportunities for earlier discussion of GWG.

Recent studies of general practitioners, obstetricians and midwives demonstrate a range of views and practices regarding GWG, with less than one third of these health professionals providing GWG guidelines and many exhibiting limited understanding of the clinical significance of excess GWG [27–30]. This may be in part explained by absence of GWG and weighing protocols, limited time for consultations for discussion of multiple antenatal issues, low awareness of guidelines for weighing and GWG, limited education regarding GWG, low levels of confidence and changes in clinical guidelines and practice resulting in non-evidence based approaches to care and advice [27–30]. New models of care that promote health professionals’ discussion and implementation of GWG monitoring and guidelines are required. Parallels may be drawn from the smoking cessation literature where combining evidence based guidelines with an implementation program addressing health professional barriers resulted in an increase in evidence-based practice with some indication of improved smoking behaviour for women [31].

Pregnancy is a time of significant information seeking and knowledge acquisition [15, 26] and therefore it is not unexpected that more than half the women (55.4 %) had actively sought GWG information. This study found that first-time mothers were seven times more likely to seek GWG information than those with subsequent pregnancies. Women are more likely to rely on their own experience in subsequent pregnancies [32]. Pregnancy is one of the few life transition points where the majority of women are interested in their health and actively seek information with the health of their baby as the central focus [26]. Given that first time mothers, in particular, are seeking GWG information this phase of life offers opportunities to influence their future health and health behaviours, in addition to subsequent pregnancy weight gains.

It is positive to note that women who were obese at conception were more likely to seek GWG information given that this group is at greater risk of exceeding GWG guidelines than those who are not obese [10]. This is in line with non-pregnancy literature suggesting that people identifying as overweight or obese had a higher level of weight concern [33]. Relatedly, a thematic analysis of 400 posts made in a UK-based parenting internet forum suggested that overweight women were concerned about the impact of their weight on the growing baby [34]. Further exploration of women’s motivations to seek information, taking into account their pre-pregnancy BMI, their GWG goals and subsequent GWG could offer insights into future models to promote healthy GWG in this high risk group.

Recent literature on information seeking in pregnancy has focused on the use of electronic media, for example the internet, as the growing and most often utilised information source [35–37]. While the vast majority of women in this study used the internet to find GWG information, and many nominated it as the most important source, women, on average, collected information from more than four sources. This finding is consistent with the uses and gratification theory [22], suggesting that women have power over their media and information consumption and take an active role in interpreting and integrating media into their own lives [22, 38].

Of interest is the high proportion of women nominating media, compared with health professionals, as their most important source of GWG information. The wide range of media sources available allows women to obtain information in formats that are accessible, affordable and culturally and linguistically appropriate which may in part explain their preference for them. This study does not provide an insight into what GWG information is contained in media sources but general studies of the internet suggest that the information is varied and often lacks an evidence base [39]. Further, if health professionals are not providing information at point of care, women maybe more likely to seek information elsewhere with other research in pregnancy suggesting that media sources, such as internet and books, are viewed as objective and up to date sources [32]. Additionally, a recent study of 613 women from 24 countries found women expressed that their confidence levels significantly increased with respect to making decisions about their pregnancy after internet usage (p < 0.05) [37]. Health professionals’ reluctance to engage with technology and media sources [28] may also contribute to this divide.

Women born outside Australia nominated media sources, including the internet, as their most important source compared with health professionals. This is not surprising given the high levels of technology usage by cultural and minority groups [40]. Internet use in pregnancy is associated with high levels of health literacy and self-efficacy [41] and, given the high levels of education in the sample, it may be seen as a positive indicator of women being able to find culturally and linguistically appropriate information. There is a need for health professionals to acknowledge the use of different media for information and incorporate it into pregnancy education and healthy GWG interventions providing guidance on data retrieval, interpretation, and application of credible sources.

To our knowledge this is the first study to explore women’s GWG information seeking predictors and sources of GWG information in detail. While it was not the study’s intention to achieve population representative data, it is useful to consider the generalisability of the sample population and hence the results. The education levels of mothers in the sample were higher than seen in other studies of young mothers or pregnant women [42]. The lower response rate achieved from low educated women is consistent with literature regarding determinants of research participation [43]. Interestingly, the study participants were more likely to be born overseas (37.6 % compared with 27 %) [44] and speak another language at home (33.9 % compared 22.2 %) [45] than the general Australian population. This is curious given past literature suggests that people from minority cultural backgrounds are less likely to participate in health related research or attend antenatal clinics [46]. This may reflect the catchment of the large maternity hospital which includes areas of high migrant populations and the open access opportunities for pregnant women at the tertiary hospital. A potential limitation of the study is the reliance on the self-reported pre-pregnancy weight with the potential for recall bias, and hence, biased analyses. This is a common concern for GWG studies where more detailed assessments are not considered feasible. A further limitation involves the size of the dataset for the logistic regression analysis of women’s most important GWG source with the elimination of the family and friends category due to insufficient numbers. Further studies with a larger data set would allow more detailed analysis of this category.

## Conclusion

This first of a kind study provides new knowledge on women’s GWG information seeking and information sources. More than half of women sought GWG guidance and were more likely to consult non-clinician sources. The small numbers provided with GWG targets, and the dominance of non-clinical information sources, supports the need for GWG guidance from reputable sources. Given the high level of media usage by women, further research is required to assess the quality of content of these resources. Integration of evidence based media sources into antenatal care would augment the care of pregnant women and may serve to improve health professional and pregnant women interaction regarding healthy GWG.

## Additional file

Additional file 1: GWG information seeking outcome measures.

## Abbreviations

BMI: Body mass index
GWG: Gestational weight gain
ppBMI: Pre-pregnancy body mass index
