# Supplementary material for: Gestational weight gain information: seeking and sources among pregnant women
Source: BMC Pregnancy Childbirth. 2015 Aug 7;15:164. doi: 10.1186/s12884-015-0600-6 (PMC4528714; doi:10.1186/s12884-015-0600-6)
Supplement: Additional file 1: — GWG information seeking outcome measures. [file 12884_2015_600_MOESM1_ESM.docx]

**Additional file 1: GWG information seeking outcome measures**

|  | **Measure** | **Question type** | **Response** | **Reliability^a^** | **Source** |
| --- | --- | --- | --- | --- | --- |
| **Sought GWG information** | During this pregnancy have you looked for information (including on the internet) or asked anyone about how much weight you should gain during pregnancy? | Dichotomous | Yes/No | κ = 0.68^b^ | Original |
| **Health professional advice** | Has your doctor or midwife given you a specific weight gain suggestion or target for this pregnancy? | Dichotomous | Yes/No | κ = 0.77^b^ | Adapted from Campbell et al [2] |
|  | What weight or weight range did the doctor or midwife advise you to reach? | Open | Open | n/a^d^ | Adapted from Campbell et al [2] |
| **GWG information sources** | Where have you asked for or looked for information about weight gain during this pregnancy? | Categorical | 16 options | 78.4-100%^c^ | Adapted from Sheih et al [1] |
|  | Which is your most important source of pregnancy information? | Dichotomous | 16 options | κ = 0.59^b^ | Adapted from Sheih et al [1] |

^a .^Survey question test-retest reliability was established via repeated administration of the survey two weeks apart in a separate subsample of 38 pregnant women.

^b.^ Kappa coefficient

^c.^ range of percentage agreement

^d.^ not applicable for reliability testing.

**References**

1. Shieh C, Broome ME, Stump TE: **Factors associated with health information-seeking in low-income pregnant women**. *Women Health* 2010, **50**(5):426-442.

2. Campbell KJ, Lioret S, McNaughton SA, Crawford DA, Salmon J, Ball K, McCallum Z, Gerner BE, Spence AC, Cameron AJ *et al*: **A parent-focused intervention to reduce infant obesity risk behaviors: a randomized trial**. *Pediatrics* 2013, **131**(4):652-660.
